# Social and Seasonal Factors Contribute to Shifts in Male African Elephant (*Loxodonta africana*) Foraging and Activity Patterns in Kruger National Park, South Africa

**DOI:** 10.3390/ani11113070

**Published:** 2021-10-27

**Authors:** Kara du Plessis, Stefanie Birgit Ganswindt, Henk Bertschinger, Bruce Crossey, Michelle Deborah Henley, Mmatsawela Ramahlo, André Ganswindt

**Affiliations:** 1 Mammal Research Institute, Department of Zoology and Entomology, University of Pretoria, Private Bag X20, Hatfield 0028, South Africa; stefanie.ganswindt@up.ac.za (S.B.G.); bruce.crossey@zoology.up.ac.za (B.C.); jawi.ramahlo@up.ac.za (M.R.); andre.ganswindt@up.ac.za (A.G.); 2 Veterinary Population Management Laboratory, Section of Reproduction, Department of Production Animal Studies, University of Pretoria, Private Bag X04, Onderstepoort 0110, South Africa; henkbert@tiscali.co.za; 3 Applied Behavioural Ecology and Ecosystem Research Unit, School of Environmental Sciences, University of South Africa, Private Bag X5, Florida 1710, South Africa; michelephant@savetheelephants.org; 4 Elephants Alive, P.O. Box 960, Hoedspruit 1380, South Africa

**Keywords:** mega-herbivore, sociality, grazing, browsing, bimodal feeding, diet-switching, keystone species

## Abstract

**Simple Summary:**

African savannah elephants are able to greatly modify the vegetation around them through their foraging activities. Accordingly, studying the factors that affect elephant foraging behaviour during different seasons are important to understand their impact on the environment and will also aid in predicting how elephants might react to potential threats such as climate change and land transformation. This article aimed to reinforce current knowledge regarding elephant foraging behaviour by examining how the behaviour is affected across (a) season (wet versus dry); (b) time of day (before or after noon); (c) presence or absence of other elephants; and (d) reproductive state; for six adult elephant bulls monitored in Kruger National Park. Results indicated that elephant foraging behaviour is indeed affected by seasonal and social factors. This highlights how these animals are able to adjust their foraging behaviour during the day to aid in thermoregulation, or during different seasons to fulfil their nutritional requirements. Furthermore, this study opens the door for further research regarding how reproductive activity affects the foraging behaviour of male elephants.

**Abstract:**

African savannah elephants (*Loxodonta africana*) are well-known as ecosystem engineers with the ability to modify vegetation structure. The present study aimed to examine how male elephant foraging behaviour is affected across (a) season (wet versus dry); (b) time of day (before or after noon); (c) presence or absence of other elephants; and (d) reproductive state (musth versus no musth). Six radio-collared adult elephant bulls were observed twice per week from June 2007–June 2008 in Kruger National Park (KNP), South Africa. Using generalized linear mixed effect modeling, results indicate that elephant bulls graze more during the wet season and browse more during the dry season. To potentially offset the costs associated with thermoregulation during the heat of the day, KNP elephants spent more time foraging during the morning, and more time resting during the afternoon. Male elephants also foraged significantly less when they were associated with females compared to when they were alone or with other males. This is likely due to male–female associations formed mainly for reproductive purposes, thus impeding on male foraging behaviours. In contrast, the condition of musth, defined by the presence of related physical signs, had no significant effect on foraging behaviour.

## 1. Introduction

Herbivore foraging behaviours have direct implications for individual survival, reproduction, and overall fitness [1,2]. At the ecosystem level, effects of herbivore foraging behaviours are often reflected in the abundance, diversity, and distribution of plant species [2,3,4]. Considering the top-down pressure that herbivores can exert on plant communities (and by extension ecosystem structure), it is critically important to understand species-specific foraging preferences across habitat types. This information can assist in determining whether the presence of a specific herbivore species has a net positive, negative or negligible effect on plant communities within a particular habitat type [5,6,7]. Consequently, this may aid in informing management interventions aimed at maintaining biodiversity within specific ecosystems.

African savannah elephants (*Loxodonta africana*), hereafter referred to as “elephants”, are mega-herbivores well known for their role in ecosystem engineering [8,9,10]. Elephants perform important ecological roles, such as affecting the quality of foliage available through their foraging activities [11]. Furthermore, they modify vegetation structure, generally by decreasing the amount of woody vegetation (which can aid in preventing bush encroachment) [12,13]; and additionally aid in seed dispersal [14]. Megaherbivores, such as elephants, hippopotamus (*Hippopotamus amphibius*), giraffe (*Giraffa* spp.), and rhino (*Ceratotherium simum* and *Diceros bicornis)* also play a considerable role in regulating plant communities as they are large and, ordinarily, generalist feeders [15,16,17]. For example, Bakker et al. [5] demonstrated that larger, generalist herbivores are able to increase plant diversity in grasslands when there is high plant productivity by exerting pressure on what would otherwise become dominant plant species [5]. The presence or absence of larger herbivores can thus have considerable effects on the vegetation on which they prefer to feed, and, in turn, biodiversity overall [15,16].

There are several factors that influence herbivore abundance, distribution and foraging behaviour. These include plant defenses, which might be structural (such as thorns) or chemical, by decreasing the nutritional value and digestibility of the plant [18]. Furthermore, factors such as climate and surface water availability affect vegetation and will therefore influence herbivore foraging decisions and distributions [19,20]. Social and reproductive behaviours might also play a role in influencing subsequent feeding behaviours [20,21]. Moose (*Alces alces*) females accompanied by calves have smaller summer home ranges than females with no calves or older more mobile young [20]. Mature (10 years and older) male African buffalo (*Syncerus caffer*) forage less when found in mixed herds during their six-month mating period compared to females and younger males in the same mixed herds [21].

For elephants in particular, season plays an important role in determining the ratio of browse to graze consumed [22,23,24]. Browsing behaviours are usually more pronounced during the dry season, whilst food obtained from grazing forms the largest part of their diet during the wet season [22,24]. This trend is not exclusive for elephants, with species such as impala (*Aepyceros melampus*), also exhibiting seasonal alterations in their foraging preferences [24]. Time of day is an equally important factor to consider, as elephants are bimodal feeders, preferring to feed during the morning and the evening, and spending more time resting in the middle of the day [25,26,27].

African savannah elephants also form social groups, the composition of which can impact their daily foraging behaviour, as males and females make use of different feeding strategies [28]. Female social groups usually comprise a matriarch and related females (mostly her daughters), alongside their dependent offspring, all of whom travel together and share strong social bonds [29,30]. When males approach sexual maturity, they leave the matriarch’s group and are subsequently either found alone, or in bachelor groups with other unrelated males [31,32,33]. Male elephants are considered adults when they are between 20 to 30 years old; however, they reach their reproductive peak when they are older than 30 years and are then considered mature adults [34]. Strict social bonds between individuals in these groups are not generally observed [31,32]. Adult male elephants are usually only found together with females for reproductive purposes, or coincidentally while passing one another at a water or food source [32,35]. The reproductive period is often characterized by male elephants exhibiting a condition known as musth [36,37]. Musth is proposed to confer a reproductive advantage, as most offspring are sired by bulls that were in musth [35,38]. There are some costs associated with entering and maintaining musth, as males usually experience a declining body condition thought to be caused by an increase in time spent traveling and mate guarding; and less time foraging [35,39]. However, bulls in musth do not appear to experience significant stress [40]. Ultimately, musth plays a key role in altering elephant behaviour by increasing bulls’ overall level of aggression, particularly towards other bulls also in musth [35,37]. Musth has been found to affect not only the sexual behaviour of elephants but also their association patterns. Males in musth will travel farther and spend more time with females and less time with other males [36,39]. As a result of the role of musth in promoting behavioural changes, it is worthwhile to further investigate what other behaviours, aside from reproduction, this period may also affect. Furthermore, as little is known regarding musth’s effect on foraging, this was considered a key factor to be investigated in the present study.

With these factors in mind, this study aimed to determine how social and seasonal factors relate to changes in foraging behaviour for adult male elephants in Kruger National Park (KNP), South Africa. More specifically, the present study examined how browsing and grazing behaviours of male African savannah elephants differed across (a) seasons (wet versus dry); (b) time of day (before noon versus after noon); (c) herd sex-structure (presence or absence of other male or female elephants); and (d) male reproductive status (musth versus no musth).

This study was conducted in the KNP, which is well known for its contributions to research and management practices [41]. The elephant population in the KNP has been under a lot of scrutiny with regards to their ecological effect on vegetation structure [15]. This is due to their ability to potentially alter the vegetation composition and structure, resulting in a possible loss of biodiversity within the park [15,42,43,44]. Since 1967, the KNP elephant population was maintained at a set number of individuals (~7500) until 1994, when culling operations were ceased [42,45,46]. In 2015, the elephant population in the KNP was estimated to have reached approximately 17,000 individuals [47]. The effects elephants might have on biodiversity are not always due to their abundance, but rather due to range limitations and the resources available in their location [48]. Nonetheless, knowledge regarding the factors that may influence male elephant foraging behaviours in the KNP, the largest game reserve in South Africa (which houses the country’s largest elephant population), could be helpful to better understand how male elephants affect their surrounding environment seasonally. Consequently, such knowledge can be beneficial in predicting potential future challenges faced by the species regarding more rapid changes in climate and land transformation.

## 2. Materials and Methods

### 2.1. Ethical Clearance

This research project commenced with the permission of the University of Pretoria Animal Ethics Committee (AEC) (Ethical clearance number: V012/06), as well as the South African National Parks’ Conservation Services.

### 2.2. Study Area

**Figure 1:**
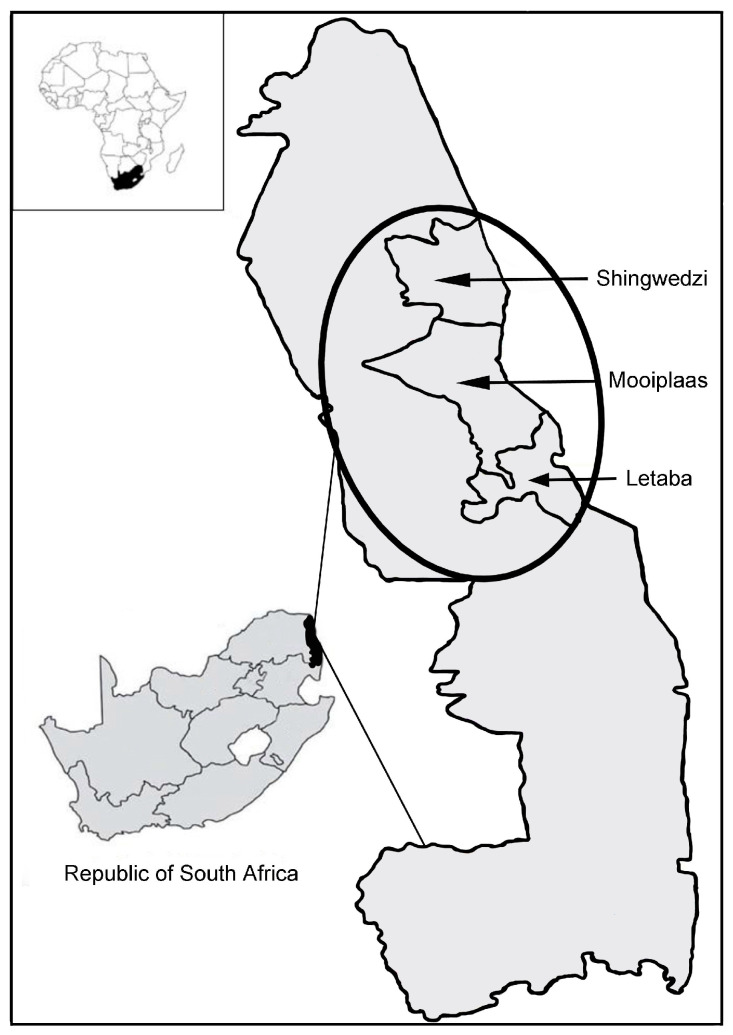
A map of Kruger National Park, South Africa. The encircled area represents the study area. Arrows indicate the sites where rainfall data were collected.

Kruger National Park (KNP) is found in the North-eastern part of South Africa and covers an area of ~20,000 km^2^. The climate and geological substrate differ throughout the park, and this provides room for a diverse array of vegetation types [49]. This allows mixed-feeders (such as elephants) to selectively forage and adjust their diets according to resource availability across seasons. In the KNP, shifts in diet due to differences in climate, or vegetation structure, can be easily studied due to the extensive body of literature documenting the ecotypes found there [23,50]. The region experiences seasonal rainfall, with the wet season occurring mostly from November to April [50]. This study was conducted in the Northern parts of the KNP and observations spanned across an area covering ~5 500 km^2^ (Figure 1). The vegetation type found in this area is predominantly savannah and shrubland, with woody cover ranging from dense to more sparse [40,51].

### 2.3. Study Animals and Behavioural Observations

Observational data were collected from six adult male African savannah elephants (referred to as Bulls 1–6) from June 2007–June 2008 (Table 1 and Table 2). Location data for each elephant was obtained from the former Save the Elephants’ Transboundary Elephant Research Program currently known as Elephants Alive, via previously fitted GPS/radio trackers. Behavioural observations took place twice per week, per elephant, after the GPS locations of each bull had been established. Each individual was observed for a minimum of 30 min per sampling session, and sampling was conducted ad libitum [52]. The behavioural observations recorded were based on whether individuals were foraging (subdivided into browsing versus grazing) or resting as well as time spent on each of these behaviours.

### 2.4. Time of Day and Season

Time of day was categorised as either before noon (05h00–12h00) or after noon (12h01–18h00). Rainfall data were also collected throughout the study period. These data were then used as a proxy to determine the season (wet versus dry) during which observations were made. Precipitation data were collected monthly at Letaba, Shingwedzi, and Mooiplaas (Figure 1). Dry seasons were recorded for the months of June–October 2007 and May–June 2008 (52–80 mm of rainfall during these periods) and a wet season occurred between November 2007 and April 2008 (378–588 mm of rainfall). This classification is in line with other studies conducted in the KNP, which indicate that the months November-April receive most of the annual precipitation [50].

### 2.5. Association and Injury

Data relating to “association” were recorded based on the sex composition of the groups within which the focal bulls were observed. Association categories were defined based on whether the elephants were observed alone, accompanied by other bulls only, or accompanied by mature females (whether other bulls were present or not). Elephants were classified as being associated with one another when they were observed to be in close proximity (not more than 10 body lengths apart) and when they moved together in a unified way during the observation period [40,51].

Injuries were noted if any swelling or signs of skin penetration were present, if individuals presented with an abnormal gait, or lifted their presumably injured foot whenever possible (especially when resting) [51]. Both Bulls 1 and 3 sustained foot injuries during the dry season of 2007. Bull 1 exhibited these injuries from June–October 2007, whilst Bull 3 showed signs of injury from August–October 2007. A priori testing revealed that injuries affected results regarding time spent grazing and browsing. As such, the individuals that presented with foot injuries (Bulls 1 and 3) were removed from analyses for the time during which they were injured.

### 2.6. Presence or Absence of Musth

**Figure 2:**
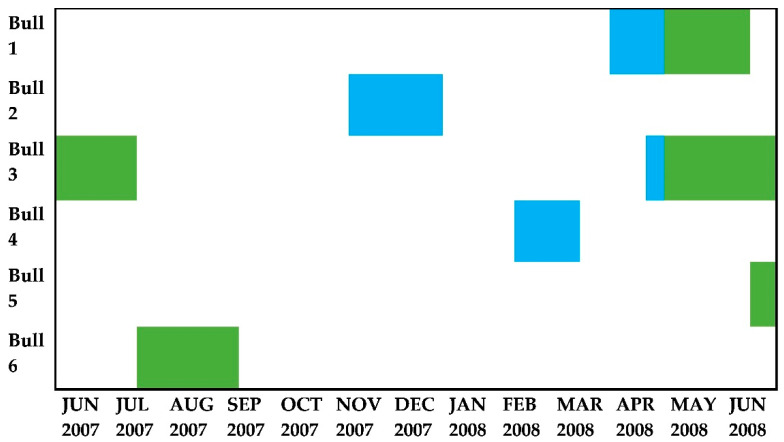
The time and duration each elephant bull experienced musth during the study. Green bars indicate periods of musth in the dry season, whilst blue bars indicate musth during the wet season.

Periods of musth were determined by looking for any visible signs of temporal gland secretion (TG) and temporal gland swelling (TGS), characteristic urine discharge (UD) accompanied by a grey to greenish discolouration of the penis and sheath, as well as the strong odour that accompanies this period [37,40]. The time and duration focal bulls experienced musth differed during the study period (Figure 2).

### 2.7. Data Analysis

Due to sampling being conducted ad libitum, time spent observing the elephants was not constant, and therefore observational data were transformed to represent a percentage of total time spent observing each individual. Generalized linear mixed-effect regression models [53] were used in order to assess the effect of time of day, season, association, and musth on the percentage time spent foraging, grazing, browsing, and resting, respectively. Individual data sets during the time injuries were observed for Bulls 1 and 3 were excluded from analyses in order to avoid any influence their injuries might have on overall results. For each model, “individual” was included as a random effect to account for any clustering of observations and to account for unequal sampling sessions per individual. To correct for autocorrelation of measurements as a result of the effect of possible individual foraging preferences between elephants, a first-order autoregressive process was also modeled by using the autocorrelation function (ACF) [54]. An autocorrelation value was estimated using a single-lag regression of the residuals for all models (Table 3). For all analyses, alpha was set at 0.05 and significance inferred at 5% and all t-statistics were obtained from modeling results. All analyses were conducted in RStudio version 4.0.3 [55]. Packages mass [56], ggplot2 [57], rstatix [58], and car [59] were used to run statistical analyses and graphically represent the data. Results are presented as boxplot graphs with the line indicating the median of the data; the box, the 25th, and 75th percentiles and the bars indicating the min and max values; whilst dots represent outliers.

## 3. Results

### 3.1. Time of Day and Season

**Figure 3:**
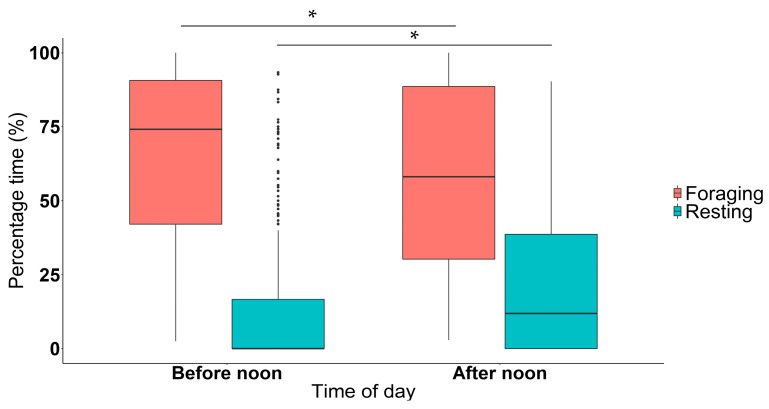
Boxplot depicting the percentage time (%) spent foraging and resting according to time of day. Number of observations = 428. * Indicates statistically significant differences between groups.

Generalized linear mixed modeling indicated the six elephant bulls foraged significantly longer before compared to after noon (*n =* 428, *df* = 417, *t*-value = 34.752, *p* < 0.050) (Figure 3). Conversely, the bulls rested significantly less before noon compared to after noon (*n* = 428, *df* = 417, *t*-value = 6.463, *p* < 0.050) (Figure 3).

**Figure 4:**
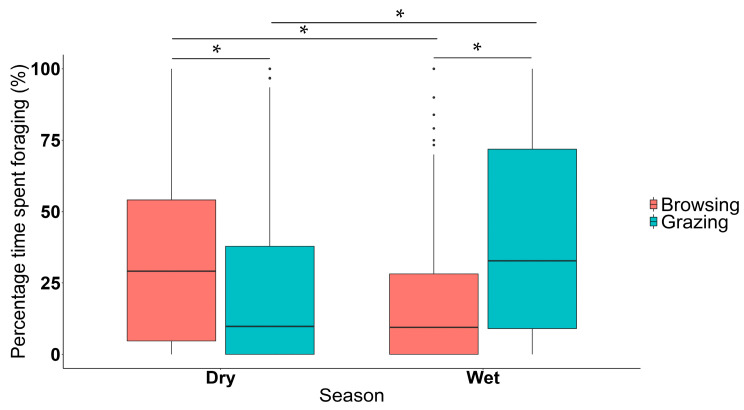
Boxplot depicting the percentage time spent foraging (%) (broken down into grazing and browsing) in the dry and wet season. Number of observations = 428. * Indicates statistically significant differences between groups.

The elephant bulls browsed significantly more during the dry season compared to the wet season (*n* = 428, *df* = 417, *t*-value = 4.587, *p* < 0.050) (Figure 4), and grazed significantly more during the wet season compared to the dry season (*n* = 428, *df* = 417, *t*-value = 16.859, *p* < 0.050) (Figure 4). In the dry season, bulls spent significantly more time browsing then grazing (*n* = 408, *df* = 401, *t*-value = 40.726, *p* < 0.050) (Figure 4). Similarly, the six bulls spent significantly more time grazing over browsing in the wet season (*n* = 448, *df* = 441, *t*-value= 39.239, *p* < 0.050) (Figure 4).

### 3.2. Association

**Figure 5:**
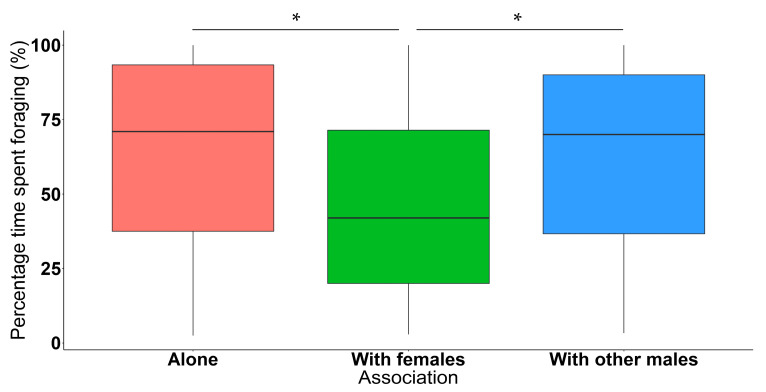
Boxplot depicting the percentage time spent foraging (%) across different associated social groups. Number of observations = 428. * Indicates statistically significant differences between groups.

**Figure 6:**
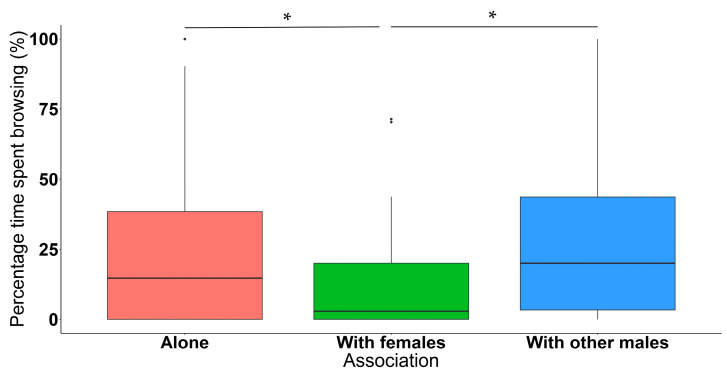
Boxplot depicting the percentage time spent browsing (%) across different associated social groups. Number of observations = 428. * Indicates statistically significant differences between groups.

Lone elephant bulls spent significantly more time foraging when compared to focal bulls who were foraging alongside females, regardless of whether other males were present or not (*n* = 428, *df* = 417, *t*-value = 33.790, *p* < 0.050) (Figure 5). When the focal bulls were in the presence of other bulls, they also spent significantly more time foraging compared to when they were associated with females (regardless of whether or not other bulls were present along with females) (*n* = 428, *df* = 417, *t*-value = 34.071, *p* < 0.050) (Figure 5). More specifically, focal bulls did not spend significantly more or less time grazing when they were alone compared to focal bulls who were grazing alongside females (*n* = 428, *df* = 417, *t*-value = 11.991, *p* = 0.809). Similarly, time spent grazing between focal bulls in an all-male group did not significantly differ compared to when they were grazing in a group with females (*n* = 428, *df* = 417, *t*-value = 11.783, *p* = 0.652). Furthermore, focal bulls spent significantly more time specifically browsing when they were alone compared to focal bulls who were browsing alongside females (*n* = 428, *df* = 417, *t*-value = 12.572, *p* < 0.050). Time spent browsing between focal bulls in an all-male group was significantly higher compared to when they were browsing in a group with females (*n* = 428, *df* = 417, *t*-value = 13.168, *p* < 0.050) (Figure 6).

### 3.3. Presence or Absence of Musth

Musth did not significantly affect the overall time bulls spent foraging (*n* = 428, *df* = 417, *t*-value = 32.723, *p* = 0.312), regardless of whether they were grazing (*n* = 428, *df* = 417, *t*-value = 12.599, *p* = 0.714) browsing (*n* = 428, *df* = 417, *t*-value = 10.689, *p* = 0.916), or resting (*n* = 428, *df* = 417, *t*-value = 10.062, *p* = 0.662).

## 4. Discussion

This study demonstrates that adult bull elephants in the KNP show shifts in their foraging behaviour and activity patterns as a result of seasonal and social factors. The data presented in this study shows that elephants spend more time foraging during the first half of the day, while spending more time resting during the latter half of the day (Figure 3). A study conducted in the KNP from August 2007 to August 2009 (around the time the current study took place) showed that ambient temperatures peaked between 12h00 to 16h00 in the afternoon [60]. Due to their large size, African elephants have a low surface-to-volume ratio, making it difficult for them to thermoregulate in an energy efficient manner [61]. To counteract this, elephants have several physiological and behavioural mechanisms which they make use of to help regulate their body temperature [62,63]. The present study highlights how elephant behaviour assists in their maintenance of homeostasis within thermoregulatory limits. This is evident in that elephants rest significantly more during the warmer part of the day (in the afternoon), while opting to forage more when it is cooler before noon. This behavioural adaptation helps avoid the metabolic costs associated with thermoregulation during the hotter periods of the day; corroborating the findings of other studies which have shown that both African and Asian elephants (*Elephas maximus*) exhibit bimodal peaks in feeding activity [25,27].

Future climate change predictions have negative implications for elephants, with the time frame for the warmer parts of the day increasing [64]. This is of particular concern as elephants will likely soon be faced with the trade-off between increasing the time they spend resting during the hotter time of the day and balancing this against vital foraging time [65]. A study concerning the daily activity patterns of eighteen African elephants in Uganda showed that they spent about 75% of their time foraging and preferred to rest in the early afternoon and in the early hours of the morning [66]. As they spend much of their time feeding, whether elephants will be able to increase their nightly foraging bouts and afternoon resting periods whilst still maintaining their required daily nutritional intake is still unclear.

As has been previously demonstrated for other elephant populations [24,67,68], this study shows that male African savannah elephants in the KNP also tend to graze more during the wet season and browse more during the dry season (Figure 4). Grass is more palatable compared to browse, with the latter often containing many secondary compounds (including higher concentrations of tannins), which also results in these plants having lower nutritional values [69]. Other mixed-feeders, such as impala and goats (*Capra* spp.), and browsers such as kudu (*Tragelaphus strepsiceros*), do not feed on shrubs containing more than 5% of condensed tannins during the wet season [69], indicating that tannin concentrations likely also affect their foraging decisions as well. Lignin concentrations in the cell walls of grasses, however, tend to increase as the dry season progresses, decreasing the grass’ nutritional value and digestibility [70,71]. It, therefore, seems that elephants may weigh the costs and benefits of grazing versus browsing as a result of their nutritional needs, and this may be one of the key drivers of repeated seasonal switches in diet [22,23,24].

Male elephants foraged significantly less when they were with females compared to when they were alone or with other males (Figure 5). Elephants usually form same-sex groups [29]; when adult male elephants are associated with females, it is usually only for short periods of time, and for reproductive purposes when females are close to or in oestrus [28,72]. As a result, mixed-sex groups with receptive females are accompanied by more males compared to groups with females which are not in oestrus [73]. A study aiming to examine the effect of the presence of a male elephant on captive female Asian elephants found that females foraged significantly less when a male was introduced [74]. The authors suggested that this might either be due to the females adapting to the male’s presence or this behaviour might indicate a social or sexual interest in the male; therefore, females possibly allocated more time to reproductive behaviours rather than foraging in the presence of males [74]. Conversely, in the presence of females, males would be expected to forage less as well, instead increasing their time spent mating and guarding females. In conclusion, it might be that free-ranging male African elephants show the same behaviour witnessed in their captive Asian elephant counterparts.

Furthermore, results indicated that male elephants specifically browsed significantly less when they were with females (Figure 6) as opposed to grazing less. It may be that this pattern of males focusing on reproductive behaviour in the presence of females, mixes with the overall seasonal pattern of more grazing in the wet season and more browsing in the dry season. Although elephants are able to reproduce year-round, studies have shown that they do show some seasonal preference for reproduction, however, exactly which factors affect their reproductive timing is still not yet fully understood [75,76]. Most of the bulls that were observed in this study also experienced musth in the dry season (Figure 2). Poole [39] found that elephants in musth spent less time foraging, and more time searching for females [39]. Therefore, the bulls might have browsed significantly less in the presence of females due to this reproduction-related behavioural shift that will be more prominent during a seasonally preferred feeding type. Similar behaviour has also been documented for mature African buffalo, which alternate between associating with mixed herds containing females and young during their six-month mating period, and herds that comprise males only [21]. In mixed herds, mature male buffalo forage less compared to females and younger adult males. This suggests a possible trade-off between reproductive activities and foraging [21].

In and out of musth foraging and activity patterns of the bulls in the current study were not significantly different, contrary to previous findings [35,39]. Ganswindt et al. [40] showed that when in musth, respective faecal glucocorticoid metabolite (fGCM) concentrations are reduced, and that musth is likely not a significant stressor for male elephants. This may indicate that in our study elephant bulls in musth still maintain their required foraging levels. Moreover, musth can also be divided into different stages; pre-musth, musth, and post-musth [51,77]. It is possible that elephant bull foraging levels may be differentially affected depending on the specific stage of musth within which the bull finds itself, and this should form the focus of future studies in this area. Previous studies have documented that when in musth, males travel long distances, and therefore decrease their time spent resting and foraging, so that they might more successfully detect females and subsequently mate with them [36,39]. When conducting a long-term study, Poole [39] found that while in musth, time spent resting and feeding significantly decreased, while the opposite was found for walking and interactive behaviours [39]. Based on the relatively small sample size presented in this study, it is suggested that further studies are required to more fully unravel whether or not musth (or different stages of musth) may have a marked effect on elephant foraging behaviour.

## 5. Conclusions

This study successfully showed how seasonal and social factors contribute to shifts in male African savannah elephant foraging behaviour. Elephants spent more time foraging during the first half of the day, opting to spend more time resting during the latter half. They tend to graze more during the wet season, and browse more during the dry season. Male elephants foraged significantly less when they were with females, and bulls in musth showed no significant differences in their foraging behaviour or overall activity patterns. These findings strengthen our understanding of adult male African savannah elephant feeding ecology and behaviour, as well as some of the possible drivers behind these factors. By improving and reaffirming knowledge surrounding these factors, we are now able to better understand how elephants influence the environment around them. This knowledge can also aid in predicting how elephants might react to potential threats such as climate change and land transformation.

## Figures and Tables

**Table 1:** Summary statistics regarding behavioural observations. All behavioural observations during the time Bulls 1 and 3 were injured were excluded.

Individual	Total Observation Time per Individual (h)	Number of Sampling Sessions per Individual	Average Sampling Session per Individual (min)	Maximum Time Spent Observing Each Individual (min)
Bull 1	27.8	45	37.1	173.0
Bull 2	39.1	76	30.9	67.0
Bull 3	40.0	78	30.7	66.0
Bull 4	36.9	68	32.5	54.0
Bull 5	36.8	62	35.6	91.0
Bull 6	52.9	99	32.0	104.0

**Table 2:** Number of observations made for each category tested throughout the study period. All behavioural observations during the time Bulls 1 and 3 were injured were excluded.

Category Recorded	Number of Observations
Time of day—before noon (05h00–12h00)	297
Time of day—after noon (12h01–18h00)	131
Wet season	224
Dry season	204
Association—alone	233
Association—with other males only	170
Association—with females	25
In musth	101
Non musth	327

**Table 3:** Autocorrelation values used in each generalized mixed effect model, to correct for autocorrelation of measurements due to the effect of possible individual foraging preferences between elephants. Response variables indicate the percentage of time spent grazing/browsing/foraging or resting during observations. * Indicates that the model produced predicted a significant effect of at least one predictor variable.

Model	Estimated Autocorrelation Value (ACF)
Foraging ~ Time of day + Season + Association + Musth *	0.087
Resting ~ Time of day + Season + Association + Musth *	0.107
Grazing ~ Time of day + Season + Association + Musth *	0.304
Browsing ~ Time of day + Season + Association + Musth *	0.178
^1^ Foraging ~ Time spent grazing versus browsing (wet season) *	0.344
^1^ Foraging ~ Time spent grazing versus browsing (dry season) *	0.137

^1^ Foraging type comprised grazing or browsing, therefore these models aimed to test how browsing and grazing would differ between groups in wet season versus the dry.

## Data Availability

Data available in a publicly accessible repository. The data presented in this study are openly available in the University of Pretoria (Figshare) research repository and can be found at https://doi.org/10.25403/UPresearchdata.15105591.v1 (accessed on 21 August 2021).
